# Assessing PFAS total in landfill leachate through multiple extraction methods and fluorine mass balance

**DOI:** 10.1007/s00216-026-06553-8

**Published:** 2026-06-04

**Authors:** Sofia Levalier, Viktor Sjöberg, Leo W. Y. Yeung, Anna Kärrman

**Affiliations:** Man-Technology-Environment (MTM) Research Centre, School of Science and Technology, Örebro University, SE-701 82 Örebro, Sweden

**Keywords:** Solid-phase extraction (SPE), Combustion ion chromatography (CIC), Ultra-short chain, PFOS, Extractable organofluorine (EOF), Mass spectrometry

## Abstract

Comprehensive quantification of per- and polyfluoroalkyl substances (PFAS) and extractable organofluorine (EOF) for assessing PFAS total in complex environmental matrices requires extraction methods capable of retaining chemically diverse fluorinated compounds. In this study, the performance of three solid-phase extraction (SPE) sorbents (weak anion exchange (WAX), weak cation exchange (WCX), and hydrophilic-lipophilic balance (HLB)) was comprehensively evaluated for the extraction of selected targeted PFAS and inorganic anions and determination of extractable fluorine from landfill leachate. Recovery experiments covering multiple PFAS classes, including PFCA, PFSA, zwitterionic PFAS, and bis-FASI, together with inorganic fluorinated anions, showed clear sorbent-dependent selectivity. WAX provided the most consistent recoveries across compound classes and yielded comparable concentrations of Σ_33_PFAS + BF_4_^−^ and EOF, indicating retention of a broad fraction of fluorinated compounds and a closed fluorine mass balance. In contrast, WCX resulted in lower EOF concentration and a negative fluorine mass balance, attributed to matrix composition that emerged as a critical factor controlling EOF recovery, with divalent cations (Ca^2+^ and Mg^2+^) causing significant EOF suppression. HLB also yielded lower EOF concentrations, primarily due to poor retention of ultra-short-chain PFAS that contributed significantly to the overall fluorine balance, and similar behavior was observed for WCX. In addition, retention of inorganic fluorinated anions indicates that EOF concentrations included both organic and inorganic fluorinated compounds. The findings reveal that both sorbent chemistry and matrix chemistry critically influence EOF quantification, thereby affecting fluorine mass balance.

**Graphical abstract:**

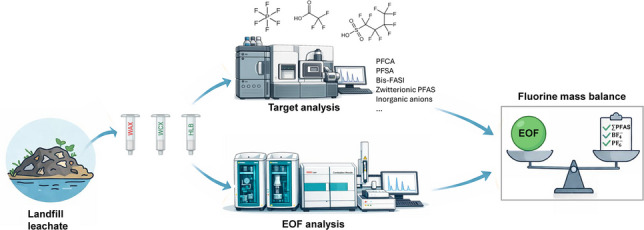

**Supplementary Information:**

The online version contains supplementary material available at 10.1007/s00216-026-06553-8.

## Introduction

Per- and polyfluoroalkyl substances (PFAS) are a broad family of synthetic fluorinated organic compounds, containing at least one fully fluorinated methyl or methylene carbon atom without any H/Cl/Br/I atom attached to it [1]. Their strong C-F bonds contribute to their chemical and thermal stability, and their hydrophilic and lipophilic characteristics contribute to their outstanding surfactant properties [2]. The combination of these properties has enabled their widespread use in industrial and consumer products, including non-stick and anti-grease coatings, stain-resistant textiles, metal plating, surfactants, hydraulic fluids, and in aqueous film-forming foams (AFFF) etc. [2, 3]. However, the same stability and resistance to degradation that made PFAS technologically valuable have also led to their persistence, global distribution, and growing concerns regarding their long-term effects on the environment and human health [4–7].

Historically, most environmental research and monitoring efforts have focused on a narrow set of legacy PFAS, particularly perfluoroalkyl sulfonic acids (PFSA) like perfluorooctane sulfonic acid (PFOS) and perfluoroalkyl carboxylic acids (PFCA) such as perfluorooctanoic acid (PFOA) [8]. Regulatory actions, such as the phase-out of PFOS and PFOA in many countries, have contributed to shifts in PFAS manufacturing toward shorter-chain analogues and fluorinated precursors [9]. However, these substitutions have not resolved the underlying challenges of their persistence, mobility, and long-term environmental load [10, 11]. Increasing attention is therefore shifting from individual PFAS to the total load of organofluorine compounds in the environment [12–14].

A critical aspect of this environmental burden arises not only from industrial production and use, but also from waste management and end-of-life pathways of PFAS-containing materials [10]. PFAS-containing consumer and industrial products enter waste streams through multiple pathways, including municipal solid waste, industrial residues, sewage sludge, AFFF-impacted waste from firefighting activities, etc. In many countries, landfilling remains a dominant disposal route for these wastes [15], resulting in the accumulation of PFAS in landfills. Over time, PFAS can migrate from waste material into landfill leachate, making landfills major secondary sources of PFAS release. This leaching may occur slowly over years or decades, meaning landfills continue to leach PFAS long after the disposal of PFAS-containing waste, and in some cases, long after the original PFAS formulations have been phased out of production [16, 17].

Landfill leachate has been shown to contain a broad spectrum of PFAS, including both precursor compounds that can degrade into intermediate and terminal end products, terminal degradation products, and fluorotelomer-based compounds originating from AFFF formulations. Previous studies demonstrate that the PFAS composition in landfill leachate is often dominated by intermediate PFAS and short-chain PFCA. For example, a study analyzing 70 PFAS in 18 landfills across the US showed that 5:3 fluorotelomer carboxylic acid (5:3 FTCA), which is a degradation product of fluorotelomer-based PFAS that can further transform into short-chain PFCA, was the dominant PFAS followed by short-chain PFCA [18]. Similar findings have been reported in other countries, where fluorotelomer-derived compounds and other polyfluorinated precursors are frequently detected [16, 19–21]. In addition, emerging PFAS classes such as bis-perfluoroalkyl sulfonimide (bis-FASI) have recently been identified in landfill leachate, highlighting new industrial sources linked to lithium-ion battery and fluoropolymer waste [22]. Other studies have detected zwitterionic PFAS (fluorotelomer betaines) in soil, groundwater, drinking water, and surface water [23–25]. Despite limited data on zwitterionic PFAS such as fluorotelomer betaines (FTB) and fluorotelomer sulfonamide betaines (FTSAB) in landfill leachate, their occurrence in landfill relevant wastes suggests a potential contribution to the total PFAS burden. Furthermore, reported PFAS profiles and concentrations vary widely between landfills within the same country and across different geographic regions because of differences in waste composition, waste age, industrial inputs, and historical use of PFAS-containing products, such as AFFF. This variability highlights the chemical complexity of landfill leachate and suggests that fluorinated compounds beyond conventional PFAS may also be present. Among these are inorganic fluorinated anions such as tetrafluoroborate (BF_4_^−^) and hexafluorophosphate (PF_6_^−^), commonly used as counter-ions in ionic liquids in different industrial applications [26, 27]. These compounds have been reported in wastewater and other environmental matrices and have been shown to contribute significantly to the measured EOF [28–30]. These findings highlight the importance of a broad screening including fluorinated compounds beyond conventional PFAS, to obtain a comprehensive understanding of the total PFAS burden and provide a foundation for improved environmental monitoring, risk assessment, and PFAS management.

Given the presence of structurally diverse fluorinated compounds, including inorganic anions, PFAS (anionic, zwitterionic, and neutral precursors), extracting and quantifying them in leachate using one single method remains analytically challenging. To overcome that, several analytical strategies have been developed as complementary approaches to the conventional targeted liquid chromatography tandem mass spectrometry (LC-MS/MS), which remains the main analytical approach for PFAS determination that is supported by standardized methods for water [31]. Among these, extractable organofluorine (EOF) determined by combustion ion chromatography (CIC) after appropriate sample preparation provides quantitative measures of total extractable organofluorine, integrating both known and unknown fluorinated species [32–34]. However, EOF represents a defined fraction that depends on extraction conditions and does not include the total fluorine originally present in a sample. Another approach is oxidative conversion that is a widely employed method to estimate oxidizable precursor contributions by converting them into measurable perfluoroalkyl acids (PFAA) [35]. In addition, high-resolution mass spectrometry (HRMS)-based suspect and non-target screening serve as supplementary tools enabling the detection of novel PFAS in the absence of certified reference standards [36–38]. Each of these methods has strengths and limitations in terms of selectivity, matrix applicability, and quantification, highlighting the need to combine different methods to cover a broad range of PFAS. However, regardless of the methods applied, to assess PFAS total in complex environmental samples, comprehensive sample extraction is needed. Solid-phase extraction (SPE) is a commonly employed method for the extraction and concentration of PFAS from landfill leachate [16, 36, 39].

The diverse chemical structures of PFAS including variations in chain length, functional groups, and polarity make it challenging to extract them all effectively using a single extraction method. Weak anion exchange (WAX) and hydrophilic-lipophilic balance (HLB) cartridges have been the most widely used sorbents for PFAS extraction from landfill leachate for both target and suspect analysis [36, 39, 40]. For EOF analysis in aqueous matrices, however, WAX has been the most frequently used sorbent [41, 42], while the use of HLB and weak cation exchange (WCX) remains limited. Despite their widespread use in PFAS analysis, comparative assessments of these sorbents particularly in the context of comprehensive fluorine mass balance have not been reported. To the best of our knowledge, no studies have systematically evaluated their performance for simultaneous PFAS and EOF extraction from landfill leachate.

This study aimed to evaluate and compare the performance of three SPE sorbents (WAX, HLB, and WCX) for EOF analysis and to assess the total PFAS load in landfill leachate, by comparing sorbent performance based on EOF, targeted PFAS recovery, and fluorine mass balance. EOF was determined by combustion ion chromatography (CIC), targeted PFAS were analyzed using LC-MS/MS and SFC-MS, and total fluorine (TF), fluoride (F^−^), BF_4_^−^, and PF_6_^−^ were measured to characterize the fluorine composition of the samples and assess their contribution to the overall fluorine balance.

## Method

### Chemicals

Methanol (LC-MS grade) (≥ 99.9%), ammonium hydroxide (25%), nitric acid (65%), and formic acid (98–100%) were purchased from Fisher Scientific. Ammonium acetate (LC-MS grade), calcium nitrate tetrahydrate (99%), and fluoride standard solution were obtained from Merck. Ultrapure water (18.2 MΩ-cm) was produced in-house using a Millipore water purification system. Weak ion-exchange (WAX) (6 cc, 500 mg, 30 µm), weak cation-exchange (WCX) (6 cc, 500 mg, 30 µm), and hydrophilic-lipophilic balance (HLB) (6 cc, 500 mg, 30 µm) cartridges were obtained from Waters Corporation.

The list of native and isotopically labeled PFAS and inorganic anions standards including full names and abbreviations is provided in Tables S1 and S2 respectively.

### Extraction method

An overview of the extraction method of PFAS, BF_4_^−^ and PF_6_^−^ for EOF and target analysis illustrated in Figure S1. Sample pretreatment and extractions were adapted from previously published methods with some modifications [31, 32, 43–45].

For SPE extraction, samples were extracted for both EOF and recovery experiments. The EOF samples were extracted without the addition of isotopically labeled extraction standards and then split after extraction into two fractions. The first fraction was analyzed for EOF by CIC and the second fraction was spiked with isotopically labeled extraction standards for quantification of PFAS, BF_4_^−^, and PF_6_^−^. For the recovery experiment, landfill leachate samples were spiked with isotopically labeled extraction standards prior to extraction.

#### Pretreatment

Landfill leachate from three different landfills in Sweden collected in 2017 and stored at −20 °C were pooled and 50 mL aliquots were used for extraction. The sample pH was adjusted before extraction; pH 2 for WAX using nitric acid, pH 9 for WCX and pH 7 for HLB using ammonium hydroxide (NH_4_OH) (see SI). The samples for recovery experiment (*n* = 3 for each sorbent) were spiked with 2 ng of each isotopically labeled extraction standard (*n* = 20) (Table S2) and then extracted using solid-phase extraction. For EOF and mass balance, the samples (*n* = 3 for each sorbent) were not spiked with mass labeled standards prior to extraction.

To evaluate the removal efficiency of inorganic fluoride during extraction, triplicates of landfill leachate for each sorbent were spiked with 15 mg/L sodium fluoride (NaF) prior to extraction.

#### SPE condition and loading

In brief, WAX and WCX sorbents were conditioned with 6 mL 0.1% ammonium hydroxide (NH_4_OH) in methanol and 2% formic acid (HCOOH) in methanol respectively, followed by 6 mL methanol and 6 mL water. HLB cartridges were conditioned with 6 mL methanol followed by 6 mL water, and then the samples (50 mL) were loaded onto the cartridges.

#### SPE wash and elution

The EOF samples were subjected to cartridge-specific washing steps optimized for the removal of inorganic fluoride (IF). Different washing solutions and volumes were evaluated, and based on the results (Figure S3), the following procedures were selected: 20 mL 0.01% NH_4_OH in water followed by 10 mL water for WAX, 30 mL water for HLB, and 20 mL 0.01% HCOOH in water followed by 10 mL water for WCX. Then followed 6 mL acetate buffer at pH 4 and pH 7.5 for WAX and WCX respectively. All cartridges were dried under vacuum for 30 min and eluted with 6 mL methanol. WAX and HLB cartridges were further eluted with 6 mL 0.1% NH_4_OH in methanol, whereas WCX cartridges were further eluted with 6 mL 2% HCOOH in methanol.

Samples used for recovery experiment, and for the quantification of PFAS, BF_4_^−^ and PF_6_^−^ were extracted using the same SPE protocol with some modifications. First, these samples were not subjected to IF removal washing steps and a wash with 20% methanol in water was included after buffer addition.

### Instrumental analysis

#### ICP-MS

Elemental analyses were performed to complement fluoride and EOF measurement and to provide additional characterization of landfill leachate, using Inductively Coupled Plasma Mass Spectrometry (ICP-MS) (Agilent 7500cx, Japan) equipped with a MicroMist nebulizer and a Scott-type double-pass spray chamber cooled to 2 °C. Sample injection was performed using a peristaltic pump and 0.89 mm i.d. tubing. Carrier and makeup gas flows were optimized to 0.9 and 0.2 L min^−1^, respectively, to maximize signal-to-noise ratios. Plasma power was set to 1500 W and the sampling depth to 8 mm. Plasma position and detector performance were optimized prior to each analytical sequence. Daily tuning and mass calibration were conducted using a 10 µg L^−1^ solution of Li, Co, Y, Ce, and Tl. Samples were introduced using a two-step procedure (60 s at 0.50 rps followed by 20 s at 0.10 rps for stabilization). Between samples, a three-step rinse was applied (0.50 rps): probe rinse with deionized water (40 s), system rinse with deionized water (40 s), and finally system rinse with 1% HNO_3_ (60 s).

#### ISE and IC

Free fluoride was determined by an ion chromatograph (Metrohm IC, Switzerland) equipped with a guard column and an anion-exchange column (Dionex AG12A and AS12A, 4 × 200 mm, respectively). A carbonate/bicarbonate eluent (2.7 mM/0.3 mM) was used as the mobile phase at a flow rate of 1.0 mL min^−1^. Deionized water and 73 mM sulfuric acid were used as regenerant solutions for the suppressor.

In addition to ion chromatography, fluoride was also determined using an ion-selective electrode (ISE) (Metrohm 6.0502.150) according to EPA Method 9214 [46]. A TISAB (Total Ionic Strength Adjustment Buffer) solution was prepared containing NaCl (58.4 g L^−1^), glacial acetic acid (57.5 mL L^−1^), and EDTA (50 g L^−1^), and adjusted to pH 5.5 using 1 mol L^−1^ NaOH. For analysis, 20 mL of TISAB was mixed with15 mL of deionized water and once the potentiometer was stable, 5 mL of sample was added. The fluoride ISE and Ag/AgCl reference electrode (Metrohm 6.0750.100) were immersed in the solution under constant stirring. The potential was recorded using a Metrohm 914 potentiometer.

#### LC/SFC-MS/MS

The identification and quantification of short- and long-chain PFAS were performed with an Acquity ultra performance liquid chromatograph (UPLC) coupled to a Xevo TQ-S tandem mass spectrometer (MS/MS) both in positive and negative mode. (Waters Corporation, Milford, US). The separation was achieved by a C18 BEH column (2.1 × 100 mm, 1.7 μm), and the mobile phases consisted of 2 mM ammonium acetate (NH_4_Ac) in 100% methanol (MeOH) and a mixture of Milli-Q water:MeOH (70:30) with 2 mM NH_4_Ac. For ultra-short chain PFAS and inorganic anions (BF_4_^−^ and PF_6_^−^), the identification and quantification were achieved using Acquity Ultra Performance Convergence Chromatograph coupled to Xevo TQ-S micro MS/MS (Waters Corporation, Milford, MA, USA) in negative mode, for separation a Torus DIOL column (3.0 mm × 150 mm, 1.7 μm) was used. The mobile phases consisted of 0.1% NH_4_OH in MeOH and CO_2_. Additional instrument parameters are provided in Tables S3–S5.

#### CIC

The EOF analysis was carried out using a combustion ion chromatography (CIC) system which consists of an autosampler and combustion module (Analytik, Jena, Germany), a 920-absorber module, and an ion chromatograph (930 Compact IC Flex, Metrohm). For the separation, an ion-exchange column (Metrosep A Supp 5–150/4) was employed, and the eluent was a carbonate buffer solution (3.2 mM sodium carbonate and 1 mM sodium bicarbonate). The injection volume was 100 µL and the samples were combusted at 1050 °C.

### Quantification and quality control (QC)

#### Fluoride

Quantification of magnesium and calcium (calibration range Mg: 0.01 µg/L–1000 µg/L, Ca: 1 µg/L–10,000 µg/L) was performed by external calibration using diluted Merck multi-element standard solution 10580. Dilution of samples and standards was performed using 1% nitric acid. Rhodium (Rh) was added as internal standard to all samples and standards. LODs were defined as three times the standard deviation of the blank projected onto the calibration curve. Recalibration was carried out after approximately 40–60 samples.

For quantification of free fluoride using IC, an external calibration (0.1–10 mg L^−1^) was performed using standards prepared by dilution of a 1 g L^−1^ sodium fluoride stock solution. All dilutions were made with ultrapure water and injected together with the sample. An external calibration (0.1–10 mg L^−1^) was used for ISE using standards prepared by dilution of a 1 g L^−1^ sodium fluoride stock solution. All dilutions were made with ultrapure water.

#### Target PFAS, BF_4_^−^, and PF_6_^−^

For each extraction, procedural blanks (*n* = 3) and QC samples (*n* = 3) for target analysis were included. Blanks and QC made from ultrapure water were spiked with 2 ng each isotopically labeled extraction standard (*n* = 20) and QC samples were spiked with 2 ng native standards (*n* = 60) (Tables S1 and S2).

The limit of detection (LOD) and quantification (LOQ) were determined for all target PFAS compounds. The LOD was set to the average blank concentration plus three times the standard deviation, and the LOQ was set to the average of the blanks plus ten times the standard deviation. In cases where no peaks were observed in the procedural blanks, the lowest point of the calibration curve was set as the LOQ. The EOF fraction spiked with isotopically labeled standard after extraction was used to quantify and estimate the contribution of PFAS, BF_4_^−^ and PF_6_^−^ to EOF. The quantification was carried out by isotope dilution using native and isotopically labeled standards. The reported concentrations were not recovery corrected. For compounds lacking corresponding isotopically labeled standards, the quantification was made using a standard with the closest retention time and/or similar structure. The recovery of zwitterionic PFAS, bis-FASI, and inorganic anion was evaluated by spiking 2 ng of the native compounds to landfill leachate prior extraction (*n* = 2). The spiked samples were then extracted together with unspiked samples as described previously. Recovery was calculated as the ratio of the measured area for the spiked sample to the sample spiked after extraction. A recovery between 50 and 120% was considered acceptable, while values below 50% or above 120% were interpreted as poor.

#### EOF and fluorine mass balance

For EOF and TF analysis, procedural blanks (*n* = 3) were included for all extractions, and combustion blanks were run before and after each sample. The method detection limit (MDL) was set to average concentrations of the blanks plus three times the standard deviation. For quantification, an external five-point calibration curve ranging from 50 to 2000 ng of F/mL made of PFOA potassium salt was used.

To evaluate the contribution of quantified PFAS to the measured EOF, procedural blank values were subtracted from all EOF results prior to further calculations. Concentrations of individual target PFAS, BF_4_^−^ and PF_6_^−^ were subsequently converted to fluorine-equivalent concentrations using the following formula:

$${C}_{F}=\frac{{C}_{PFAS}\times 19\times {n}_{F}}{{MW}_{PFAS}}$$

where ${C}_{PFAS}$ represents the measured concentration of each compound (ng/L), 19 is the atomic number of fluorine (g/mol), ${n}_{F}$ is the number of fluorine atoms in the molecule, and ${MW}_{PFAS}$ is the molecular weight of the respective PFAS (g/mol).

Fluorine-equivalent concentrations were calculated for all quantified target compounds and summed to obtain the total fluorine concentration from target PFAS and inorganic anions (∑_33_PFAS + BF_4_^−^ + PF_6_^−^). This sum was directly compared with the EOF values measured, as both were expressed in fluorine equivalents.

## Results and discussion

### Free fluoride and total fluorine

Free fluoride (F^−^) concentration (average ± standard deviation) in leachate sample measured by ISE (*n* = 3) was 1023 ± 0.03 ng/mL and 1150 ng/mL by IC (*n* = 1), whereas the TF concentration (inorganic + organofluorine) in the untreated leachate measured by CIC (*n* = 3) was 963 ± 21 ng/mL. The 95% confidence interval of CIC measurement overlapped with ISE results, indicating that the difference between free fluoride and TF was not statistically significant and was within analytical uncertainty. These results support the conclusion that free fluoride is the major fluorine species present in the leachates, whereas organic fluorine represents a comparatively smaller fraction Table 1.

**Table 1:** Average concentrations of free fluoride (F^−^) and total fluorine (TF) with standard deviation in untreated landfill leachate measured by three different methods

Method	F^−^ (ng/mL)	TF (ng/mL)
ISE	1023 ± 0.03	$-$
IC	1150	$-$
CIC	$-$	963 ± 21

### Fluorine mass balance

**Fig. 1:**
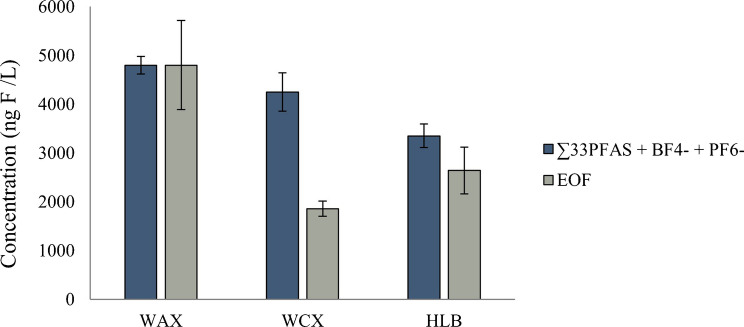
Concentrations of ∑_33_PFAS + BF_4_^−^ + PF_6_^−^, and EOF (ng F/L) in landfill leachate extracted using WAX, WCX, and HLB sorbents. Error bars represent the standard deviation of triplicate extractions

The fluorine mass balance of the leachate sample determined by comparison of EOF with target fluorine containing compounds (target PFAS, BF_4_^−^, and PF_6_^−^) varied among the different SPE sorbents (Fig. 1). The sum of quantified PFAS, together with selected inorganic anions (Σ_33_PFAS + BF_4_^−^ + PF_6_^−^), was highest for WAX (4803 ng/L F), followed by HLB (3721 ng/L F) and then WCX (4252 ng/L F). For WAX, the EOF and ΣPFAS + BF_4_^−^ + PF_6_^−^ concentrations were comparable indicating a closed fluorine mass balance. In contrast, WCX and HLB showed lower EOF concentrations relative to Σ_33_PFAS + BF_4_^−^ + PF_6_^−^, resulting in a negative mass balance. For HLB, the contribution of target fluorine explained 124% of the EOF, exceeding the expected mass balance. However, a Welch’s *t*-test showed no significant difference (*p* > 0.05) between EOF and Σ_33_PFAS + BF_4_^−^ + PF_6_^−^ concentrations, meaning that the difference is within the measurement variability. The Σ_33_PFAS + BF_4_^−^ + PF_6_^−^ concentration for WCX explained 228% of the EOF concentration, exceeding the mass balance by 128% which is above the previously reported analytical uncertainties.

A Welch’s *t*-test comparing EOF and Σ_33_PFAS + BF_4_^−^ + PF_6_^−^ concentrations for WCX showed a statistically significant difference (p < 0.05), indicating that organofluorine was not recovered to the same extent represented by the target compounds.

WCX sorbents contain weakly acidic carboxylic functional groups that are negatively charged under typical extraction conditions, enabling electrostatic interactions with positively charged and zwitterionic compounds including metal ions such as Mg^2+^ and Ca^2+^. To investigate the underestimation of EOF in the WCX extracts, the concentration of these metal ions was measured in untreated leachate and after WCX extraction (*n* = 3) using ICP-MS (Fig. S2). The concentrations of Mg^2+^ and Ca^2+^ in WCX extracts (1.07 $\times$ 10^5^ ng/mL and 1.22 $\times$ 10^5^ ng/mL respectively) were comparable to those measured in the raw leachate (1.20 $\times$ 10^5^ ng/mL and 1.72 $\times$ 10^5^ ng/mL) indicating that these cations were retained and co-eluted in the extract. EOF measured by CIC is determined by combusting the sample at 1050 °C, converting fluorine compounds to HF, which is trapped, dissociated to F^−^, and quantified by IC [47]. Since fluoride has high charge density and strong electronegativity, it can form stable complexes with metal cations. It is therefore hypothesized that fluoride released during combustion in the presence of Mg^2+^ and Ca^2+^ may have led to the formation of stable metal fluorides, such as CaF_2_ and MgF_2_, and these low-volatility fluoride species may limit quantitative conversion of fluorine to HF during CIC analysis. Both Mg^2+^ and Ca^2+^ play an important role in the fate of fluoride during combustion [47–49] and thermal treatment studies have reported that calcium promotes fluorine retention in solid phases rather than release as HF [50].

**Fig. 2:**
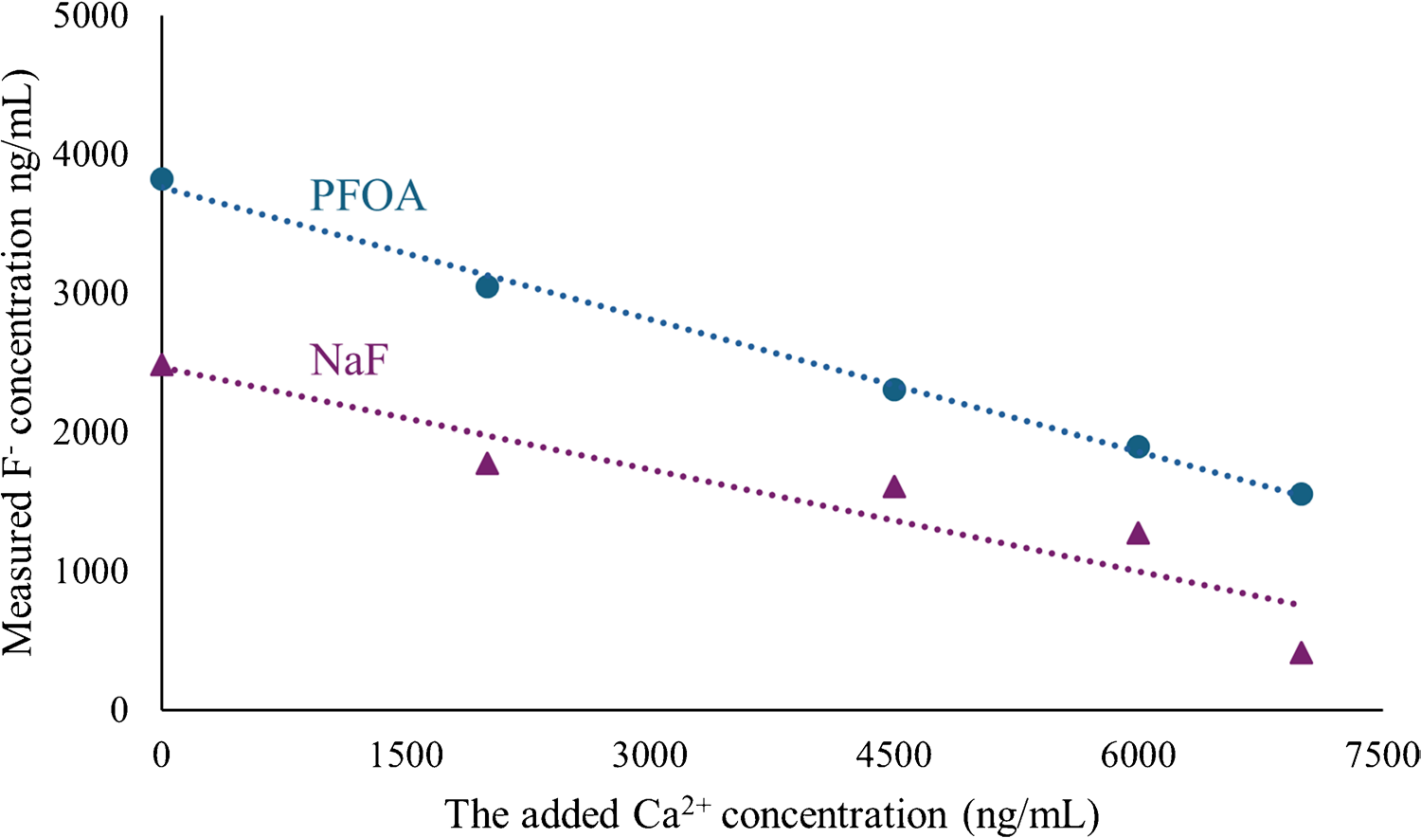
The effect of adding Ca^2+^ (0–7000 ng/mL) on the measured fluoride (ng/mL) of PFOA (3900 ng/mL) and NaF (2500 ng/mL) standards

To further confirm the interaction of Ca^2+^ with F^−^, PFOA and NaF standards (in methanol) containing a concentration of 3900 ng/mL and 2500 ng/mL respectively were spiked with increasing Ca^2+^ concentration (0–7000 ng/mL) allowing evaluation of the effect of calcium on the measured fluorine (Fig. 2). At the highest Ca^2+^ concentration (7000 ng/mL), the fluorine concentration was suppressed by approximately 60% for PFOA and 80% for NaF relative to the standards with no fluorine. This negative correlation clearly demonstrates that elevated concentrations of calcium result in reduced fluorine recovery during combustion, resulting in EOF underestimation.

### Contribution of Target PFAS, BF_4_^−^, and PF_6_^−^ to EOF

**Fig. 3:**
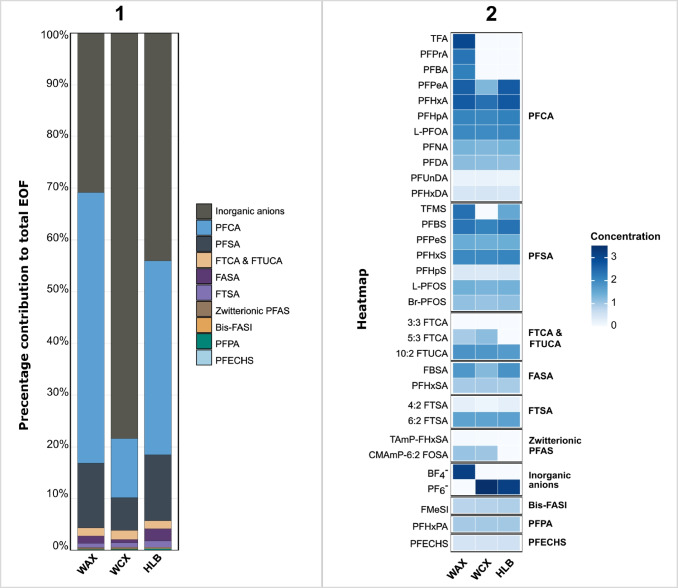
Stacked bar graph (1) showing the relative contributions (%) of target PFAS groups and inorganic anions to EOF in leachate for different SPE sorbents. The heatmap (2) showing log_10_-transformed concentration of individual PFAS, BF_4_^−^ and PF_6_^−^

Differences in PFAS profile distribution among sorbents were noted (Fig. 3). Across all sorbents, PFCA, PFSA, and inorganic anions were the major contributors to EOF. For WAX, PFCA and BF_4_^−^ contributed most to the EOF, with PFCA accounting for 52% and BF_4_^−^ for 31% of the total EOF. PFSA contributed to the total EOF with 12%, and 5% was accounted for by FTSA, FTCA, PFPA, FASA, bis-FASI, zwitterionic PFAS, and PFECHS. In contrast, for WCX and HLB, PF_6_^−^ accounted for 79% and 44%, respectively, while PFCA accounted for 11% and 38% of the total EOF. For WCX, 6% of the total EOF was accounted for by FTSA, while the remaining 4% was accounted for by FTUCA, FTCA, PFPA, FASA, bis-FASI, zwitterionic PFAS, and PFECHS.

The three sorbents showed different PFAS retention patterns that reflect their interaction mechanisms and pH conditions used during extraction. The measured EOF in WAX extracts was dominated by PFCA (C2–C10) followed by BF_4_^−^, reflecting the sorbent’s strong anion-exchange interactions. In contrast, the measured EOF in HLB extracts was dominated by mid- and long-chain PFCA (C5–C10) and PF_6_^−^, consistent with the reversed-phase retention characteristics of the HLB. Similar results were observed for WCX, for which PF_6_^−^, mid- and long chain PFCA accounted for the largest fraction of EOF, with PF_6_^−^ accounting for ~ 78%. Their retention is driven primarily by hydrophobic interactions, and in the case of PF_6_^−^, may be further enhanced by ion-pairing with metal cations present in the matrix.

Notably, these results indicate that a large portion of the extractable fluorine in the leachate sample is inorganic, meaning the EOF cannot be interpreted as “extractable organofluorine” unless inorganic anions are washed away or quantified and subtracted from “EOF”. Addition of NaF prior to extraction confirmed efficient removal of free fluoride (Fig. S3). However, NaF primarily represents free fluoride and does not fully capture the behavior of other inorganic fluorinated species during extraction. In contrast, BF_4_^−^ and PF_6_^−^ were retained by all three sorbents, demonstrating that inorganic fluorinated anions may contribute to the measured EOF.

### Recoveries of PFAS and inorganic anions

**Fig. 4:**
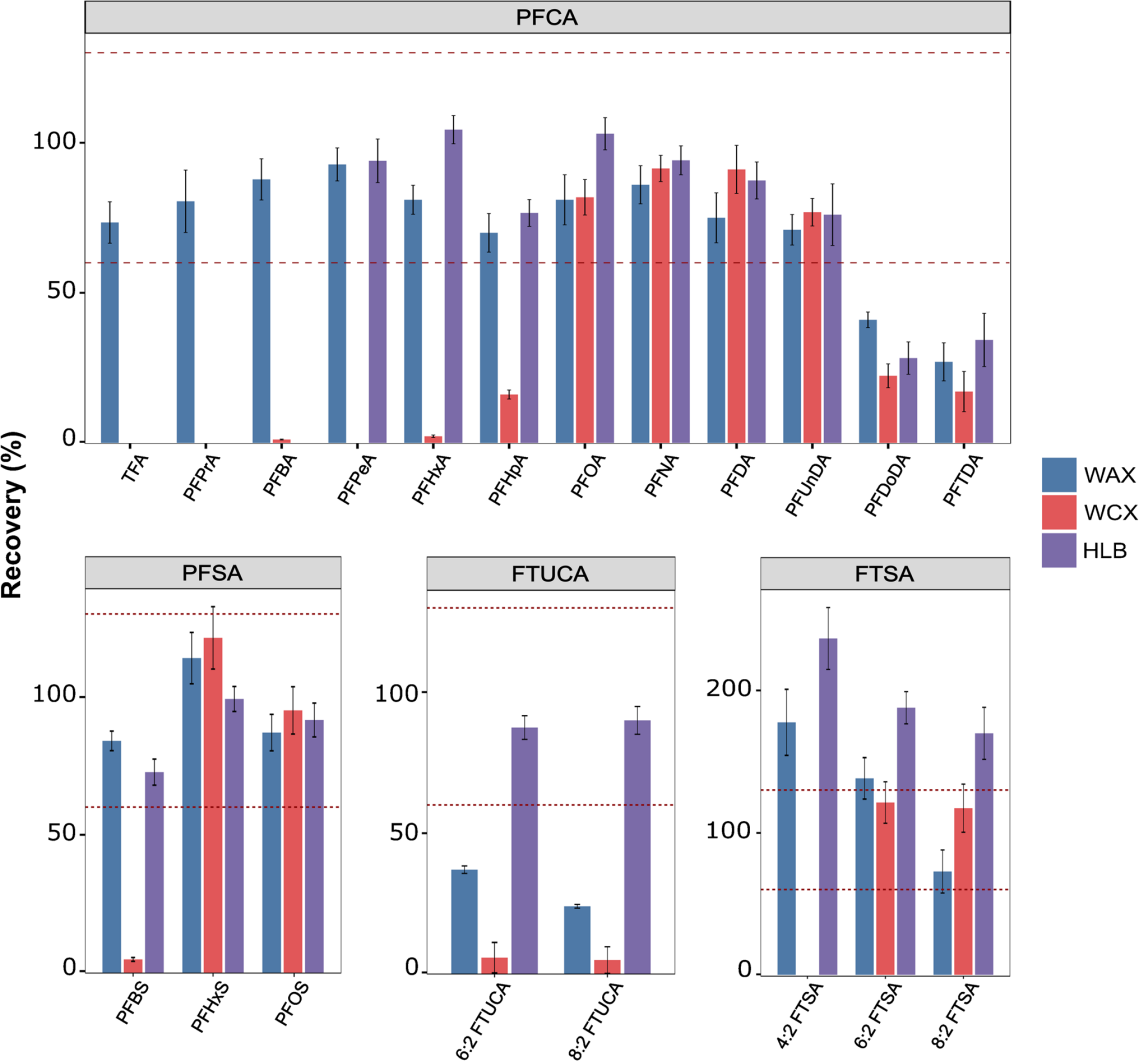
Recoveries (%) of isotopically labeled PFAS extraction standards added to leachate extracted by different sorbents, WAX, WCX, and HLB (average of *n* = 3, error bar shows the standard deviation)

The recoveries of 20 PFAS mass-labeled standards were assessed for the three different sorbents (Fig. 4). The results varied markedly across PFAS classes and sorbent types, highlighting the influence of both functional group and chain length on extraction efficiency.

The recoveries of PFCAs across the three sorbents exhibit distinct trends as a function of chain length. For WAX, recoveries were generally high for PFCAs, particularly from C2 (TFA) to C11 (PFUnDA), but decreased for chain length > C11. This could be due to the weak anion-exchange functionality, which is optimized for retaining more polar, anionic compounds. As chain length increases, the hydrophobicity of PFCA also increases, which reduces the elution efficiency and thus the overall recovery. In contrast, WCX, which employs a strong cation exchange mechanism and lacks anion-exchange capacity, showed negligible recovery for C2–C7 PFCA, indicating insufficient hydrophobic interaction for retention. However, an increased recovery was observed for C8–C11 PFCA, where retention is driven predominantly by hydrophobic interactions with the sorbent backbone. HLB exhibited more consistent and generally high recoveries for PFCA, particularly for mid- to long-chain compounds (C6–C11), due to increased hydrophobicity and stronger reversed-phase (van der Waals) interactions with the sorbent. Because HLB relies on a balanced hydrophilic-lipophilic retention mechanism, ultra-short and short-chain acids (TFA, PFPrA, and PFBA) which are highly water soluble and less hydrophobic were not retained. The recoveries for PFDoDA and PFTDA were below 50% for all sorbents.

For PFSA, the recoveries were within the acceptable range (60–120%) for PFHxS and PFOS for all the sorbents, while the short-chain PFBS exhibited low recovery on WCX. The same was observed for FTSA, where 4:2 FTSA had no retention on WCX. This could be due to the low hydrophobicity of the compounds leading to reduced interaction with the sorbent. The observed recoveries exceeding 120% for FTSA may likely be due to matrix enhancement and the lack of exactly matched isotopically labeled injection standards for these analytes. Such high recoveries have been previously reported for fluorotelomer sulfonates [51, 52].

FTUCA recoveries (6:2 and 8:2 FTUCA) were unexpectedly low for both WAX and WCX, while HLB showed good retention for both compounds. This could indicate method-specific factors affecting their retention on WAX and WCX rather than sorbent chemistry.

**Fig. 5:**
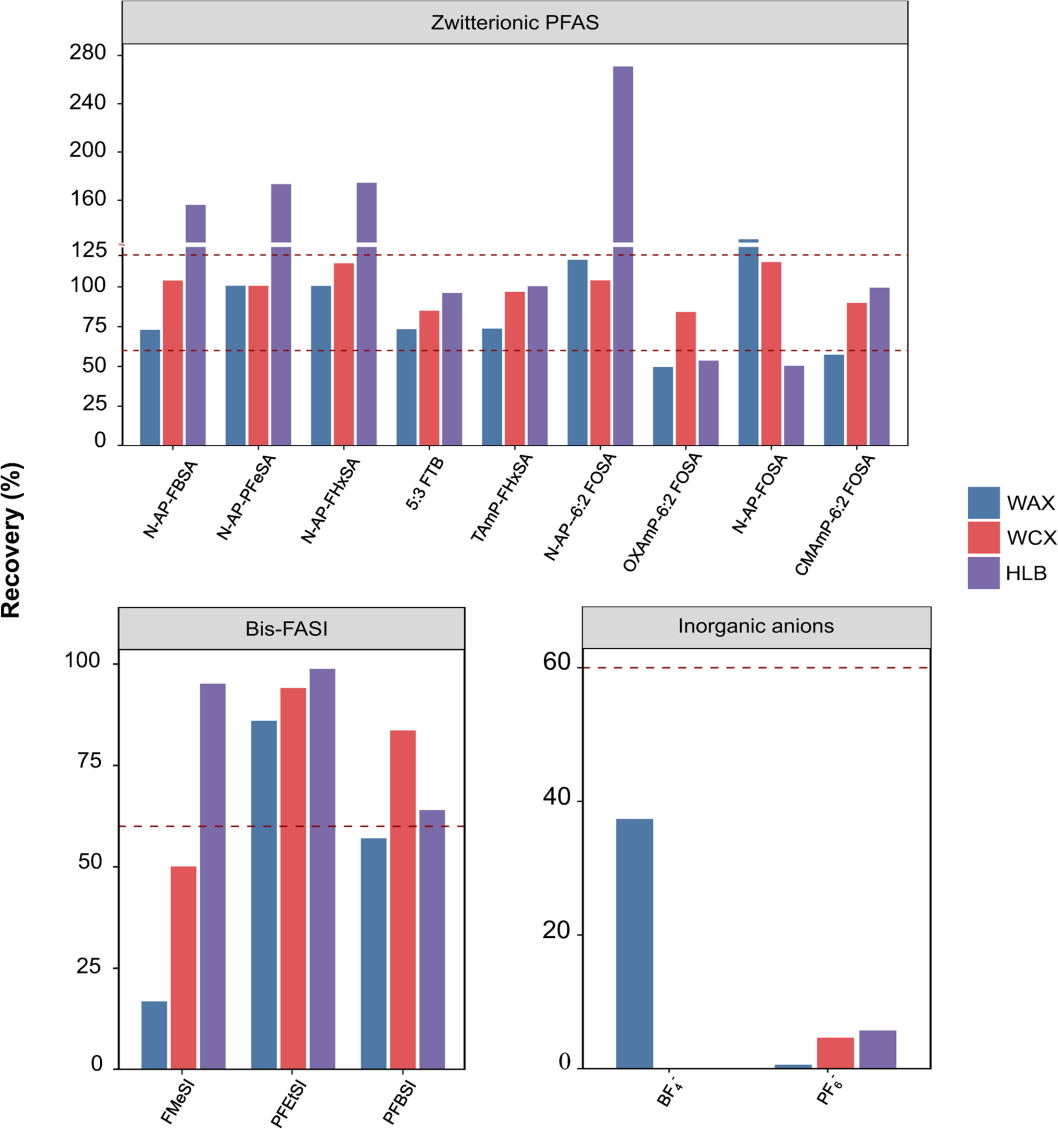
Recoveries (%) of selected native PFAS and inorganic anions added to leachate samples (*n* = 2) and extracted with different sorbents, WAX, WCX, and HLB

For compounds lacking a corresponding mass-labeled standard, pre- and post-spike experiments were conducted and the recoveries of 41 PFAS and two inorganic anions were evaluated (Fig. 5, Table S6). For zwitterionic PFAS, the recoveries varied among the sorbents, with WCX yielding acceptable recoveries for all compounds (84–116%). In contrast, WAX exhibited lower overall recoveries but remained within the acceptable range for most compounds, except for OXAmP-6:2 FOSA and CMAmP-6:2 FOSA (< 60%) and N-AP-FOSA (> 120%). However, HLB exhibited higher variability compared to WAX and WCX, with four compounds showing recoveries > 120%. The observed variation was likely driven by variability between duplicate analysis, suggesting analytical variability that likely influenced the calculated recoveries.

Bis-FASI exhibited generally acceptable recoveries across all sorbents. However, the recoveries of FMeSI obtained by WAX and WCX were below 60%, which was likely due to higher uncertainty from subtracting a relatively high concentration already present in the leachate sample compared to the added amount.

For the inorganic anions BF_4_^−^ and PF_6_^−^, the recoveries were below 60% for all sorbents. BF_4_^−^ was only retained by WAX (37%) while recoveries for HLB and WCX were negligible, and PF_6_^−^ recoveries were < 10% for all sorbents. WAX showed lower recoveries compared to HLB and WCX and previous studies have reported substantially higher recoveries for PF_6_^−^ using WAX in wastewater [29]. Based on previous studies of PF_6_^−^ hydrolysis under acidic conditions [26, 53], it was hypothesized that the low sample pH of 2 used for WAX extraction may have contributed to the observed losses. To evaluate this, PF_6_^−^ was analyzed in landfill leachate samples with (*n* = 2) and without (*n* = 2) acidification to pH 2 by direct injection. Acidified samples showed an approximately 40% decrease in PF_6_^−^ concentration (Fig. S4), indicating that sample acidification prior to WAX extraction could have contributed to the low recoveries, potentially due to acid-induced transformation such as hydrolysis.

## Conclusion

This study demonstrates that EOF analysis in landfill leachate is strongly influenced by both sorbent chemistry and matrix composition. For the specific landfill leachate investigated in this study, WAX showed the most consistent performance across the studied compound classes and comparable concentrations of Σ_33_PFAS + BF_4_^−^ and EOF within analytical variability, resulting in a closed fluorine mass balance. This indicates that the landfill leachate investigated in this study was dominated by detectable and quantifiable fluorinated species that were efficiently retained by WAX. In contrast, WCX yielded low EOF concentrations, resulting in a negative mass balance, which was attributed to the presence of divalent cations causing EOF underestimation. However, WCX may still be applicable for EOF analysis if divalent cations are removed prior to analysis, for example through precipitation or chelating clean-up steps. HLB also showed lower EOF concentration compared to WAX, likely due to the poor retention of ultra-short chain PFAS that contributed significantly to the overall mass balance, and similar results were observed for WCX.

Notably, inorganic fluorinated anions such as BF_4_^−^ and PF_6_^−^ were retained and contributed to the measured EOF, despite EOF methods being primarily designed to quantify organic fluorinated compounds. Other inorganic fluorinated anions (e.g., AsF_6_^−^ reported by a recent study [54]) may also be present and contribute to EOF in complex environmental samples and should therefore be considered.

Overall, this study demonstrates that both sorbent chemistry and matrix composition must be considered when applying combined PFAS, inorganic anions, and EOF approaches to complex environmental samples.

## Supplementary Information

Below is the link to the electronic supplementary material.

Supplementary file1 (DOCX 248 KB)

## Data Availability

All data generated during this study are included in this published article and its Supplementary Information file.
