# Supplementary material for: Assessing PFAS total in landfill leachate through multiple extraction methods and fluorine mass balance
Source: Anal Bioanal Chem. 2026 Jun 4;418(14):4575–87. doi: 10.1007/s00216-026-06553-8 (PMC13375674; doi:10.1007/s00216-026-06553-8)
Supplement: Supplementary file 1 — Supplementary file1 (DOCX 248 KB) [file 216_2026_6553_MOESM1_ESM.docx]

**Supplementary information**

Assessing PFAS Total in landfill leachate through multiple extraction methods and fluorine mass balance

Sofia Levalier^1^, Viktor Sjöberg^1^, Leo Yeung^1^, Anna Kärrman^1*^

^1^ Man-Technology-Environment (MTM) Research Centre, School of Science and Technology, Örebro University, Sweden, SE-701 82

* Corresponding author: Anna Kärrman, E-mail: anna.karrman@oru.se

**Materials and methods:**

The extraction procedure was adapted from previously published methods with modifications. For WAX, the extraction was adapted from ISO 21675:2019 [1] and the pH 2 condition was selected as a method-specific modification based on internal laboratory optimization, which showed improved recoveries of ultra-short chain PFAS under acidic loading conditions. For HLB, the extraction procedure was adapted from a previously published method using Oasis HLB cartridges, with near neutral loading conditions [2,3]. For WCX, the extraction protocol was adapted from a previously published method [4]pH 9 was applied during loading to evaluate the sorbent under alkaline conditions.

**
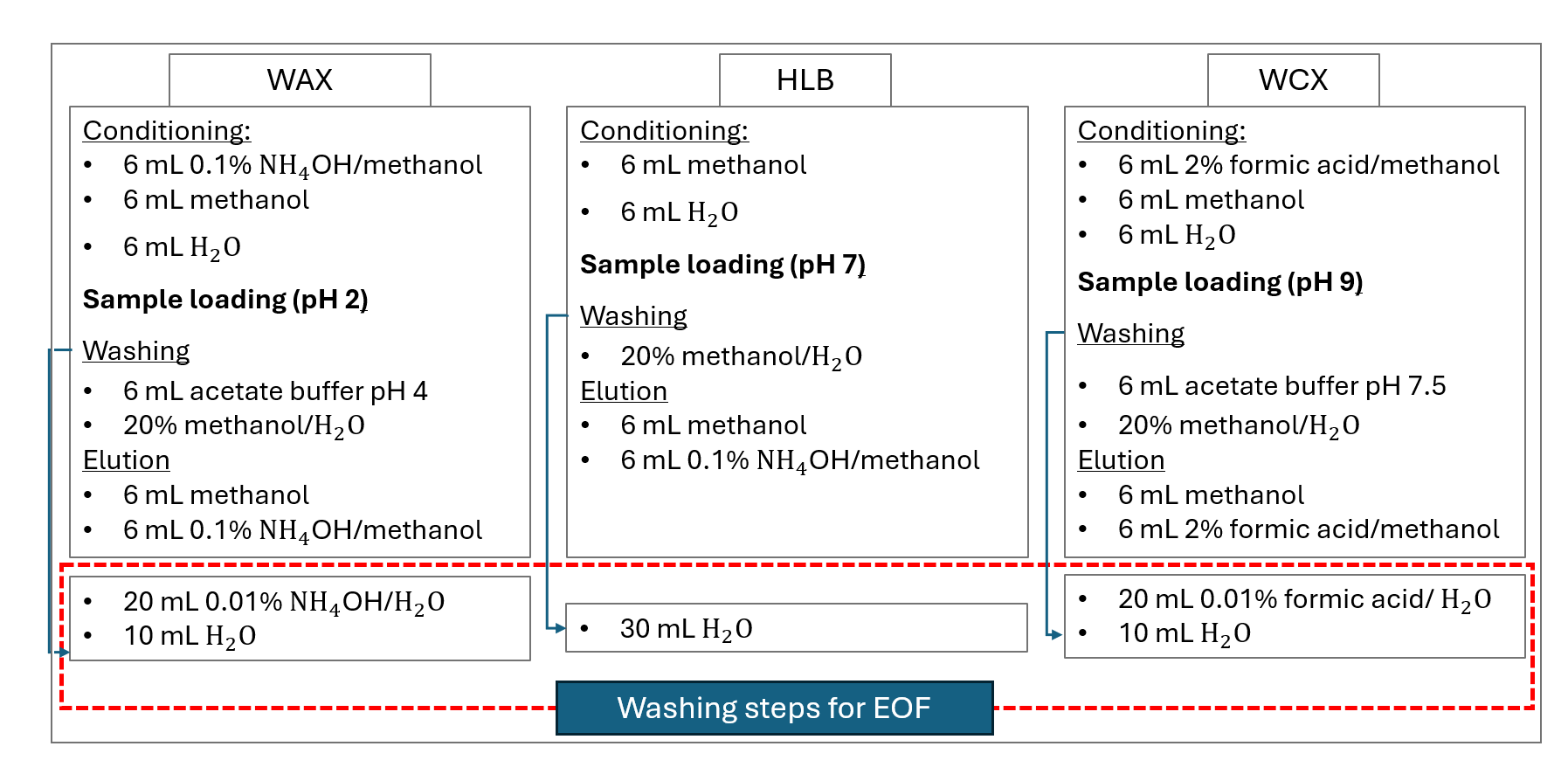
**

Figure S1: SPE-extraction scheme

Table S1: List of native standards

| Group | Acronym | Name |
| --- | --- | --- |
| PFCA | TFA | Trifluoroacetic acid |
|  | PFPrA | Perfluoropropionic acid |
|  | PFBA | Perfluorobutanoic acid |
|  | PFPeA | Perfluoropentanoic acid |
|  | PFHxA | Perfluorohexanoic acid |
|  | PFHpA | Perfluoroheptanoic acid |
|  | PFOA | Perfluorooctanoic acid |
|  | PFNA | Perfluorononanoic acid |
|  | PFDA | Perfluorodecanoic acid |
|  | PFUnDA | Perfluoroundecanoic acid |
|  | PFDoDA | Perfluorododecanoic acid |
|  | PFTrDA | Perfluorotridecanoic acid |
|  | PFTDA | Perfluorotetradecanoic acid |
|  | PFHxDA | Perfluorohexadecanoic acid |
|  | PFODA | Perfluorooctadecanoic acid |
| PFSA | TFMS | Trifluoromethanesulfonic acid |
|  | PFEtS | Perfluoroethane sulfonic acid |
|  | PFPrS | Perfluoropropane sulfonic acid |
|  | PFBS | Perfluorobutane sulfonic acid |
|  | PFPeS | Perfluoropentane sulfonic acid |
|  | PFHxS | Perflurohexane sulfonic acid |
|  | PFHpS | Perfluoroheptane sulfonic acid |
|  | PFOS | Perfluorooctane sulfonic acid |
|  | PFNS | Perfluorononane sulfonic acid |
|  | PFDS | Perfluorodecane sulfonic acid |
|  | PFDoDS | Perfluorododecane sulfonic acid |
| FTSA | 4:2 FTSA | 4:2 Fluorotelomer sulfonic acid |
|  | 6:2 FTSA | 6:2 Fluorotelomer sulfonic acid |
|  | 8:2 FTSA | 8:2 Fluorotelomer sulfonic acid |
|  | 10:2 FTSA | 10:2 Fluorotelomer sulfonic acid |
| FTCA | 3:3 FTCA | 3:3 Fluorotelomer carboxylic acid |
|  | 5:3 FTCA | 5:3 Fluorotelomer carboxylic acid |
|  | 7:3 FTCA | 7:3 Fluorotelomer carboxylic acid |
| FTUCA | 6:2 FTUCA | 6:2 Fluorotelomer unsaturated carboxylic acid |
|  | 8:2 FTUCA | 8:2 Fluorotelomer unsaturated carboxylic acid |
|  | 10:2 FTUCA | 10:2 Fluorotelomer unsaturated carboxylic acid |
| PFPiA | C6/C6 PFPiA | Bis (perfluorohexyl) phosphinic acid |
|  | C8/C8 PFPiA | Bis (perfluorooctyl) phosphinic acid |
| FASA | FBSA | Perfluorobutane sulfonamide |
|  | Me-FBSA | Methyl perfluorobutane sulfonamide |
|  | FHxSA | Perfluorohexane sulfonamide |
|  | Me-FHxSA | Methyl perfluorohexane sulfonamide |
| PFECA | HFPO-DA | Hexafluoropropylene oxide dimer acid |
| PFECHS | PFECHS | Perfluoroethylcyclohexane sulfonic acid |
| PFESA | 6:2 Cl-PFESA | 6:2 chlorinated polyfluorinated ether sulfonate |
|  | 8:2 Cl-PFESA | 8:2 chlorinated polyfluorinated ether sulfonate |
| Zwitterionic PFAS | N-AP-FBSA | N-[3-(dimethylamino)propyl]-(perfluorobutane-1-sulfonamide) |
|  | N-AP-PFeSA | Perfluoropentane sulfonamido amine |
|  | N-AP-FHxSA | Perfluorohexane sulfonamido amine |
|  | 5:3 FTB | 5:3 Fluorotelomer betaine |
|  | TAmP-FHxSA | N-[3-(Trimethylammonium)propyl]perfluoro-1-hexanesulfonamidate |
|  | N-AP-6:2 FOSA | 6:2 Fluorotelomer sulfonamide amine |
|  | OXAmP-6:2 FOSA | 6:2 Fluorotelomer sulfonamide amine oxide |
|  | N-AP-FOSA | 6:2 Fluorotelomer sulfonamide alkylbetaine |
|  | CMAmP-6:2 FOSA | 6:2 Fluorotelomer sulfonamide alkylbetaine |
| Bis-FASI | FMeSI | Bis[(trifluoromethylsulfonyl)imide |
|  | PFEtSI | Bis(perfluoroethylsulfonyl)imide |
|  | PFBSI | Bis(perfluorobutylsulfonyl)amide |
| Inorganic anions | BF_4_^-^ | Tetrafluoroborate |
|  | PF_6_^-^ | Hexafluorophosphate |

Table S2: List of isotopically labeled standards: extraction standards were spiked prior extraction and injection standards were spiked prior analysis.

| **Acronym** | **Name** | **Type of standard** |
| --- | --- | --- |
| ^13^C_2_ TFA | Trifluoro-[1,2-^13^C_2_]acetic acid | Extraction standard |
| ^13^C_3_ PFPrA | Perfluoro-[2,2,3,3,3-^13^C_3_]propanoic acid |  |
| ^13^C_4_ PFBA | Perfluoro-n-[2,3,4-^13^C_4_]butanoic acid |  |
| ^13^C_5_ PFPeA | Perfluoro-n-[3,4,5-^13^C_5_]pentanoic acid |  |
| ^13^C_5_ PFHxA | Perfluoro-n-[1,2-^13^C_5_]hexanoic acid |  |
| ^13^C_4_ PFHpA | Perfluoro-n-[1,2,3,4-^13^C_4_]heptanoic acid |  |
| ^13^C_8_ PFOA | Perfluoro-n-[^13^C_8_]octanoic acid |  |
| ^13^C_9_ PFNA | Perfluoro-n-[^13^C_9_]nonanoic acid |  |
| ^13^C_6_-PFDA | Perfluoro-n-[^13^C_6_]decanoic acid |  |
| ^13^C_7_ PFUnDA | Perfluoro-n-[1,2,3,4,5,6,7-^13^C_7_]undecanoic acid |  |
| ^13^C_2_ PFDoDA | Perfluoro-n-[1,2-^13^C_2_]dodecanoic acid |  |
| ^13^C_2_ PFTDA | Perfluoro-n-[1,2-^13^C_2_]tetradecanoic acid |  |
| ^13^C_2_ PFHxDA | Perfluoro-n-[1,2-13C2]hexadecanoic acid |  |
| ^13^C_3_-PFBS | Sodium perfluoro-1-[2,3,4-^13^C_3_]butanesulfonate |  |
| ^13^C_3_-PFHxS | Sodium perfluoro-1-[^13^C_3_]hexanesulfonate |  |
| ^13^C_8_-PFOS | Sodium perfluoro-1-[^13^C_8_]octanesulfonate |  |
| ^13^C_2_-4:2 FTSA | Sodium 1H,1H,2H,2H-perfluoro-1-[1,2-^13^C_2_]hexane sulfonate |  |
| ^13^C_2_-6:2 FTSA | Sodium 1H,1H,2H,2H-perfluoro-1-[1,2-^13^C_2_]octane sulfonate |  |
| ^13^C_2_-8:2 FTSA | Sodium 1H,1H,2H,2H-perfluoro-1-[1,2-^13^C_2_]decane sulfonate |  |
| ^13^C_2_ 6:2 FTUCA | 2H-perfluoro-[1,2-13C2]-2-decenoic acid |  |
| ^13^C_2_ 8:2 FTUCA | 2H-perfluoro-[1,2-^13^C_2_]2-octenoic acid |  |
| ^2^H_3_ N-MeFOSA | N-methyl-^2^H_3_-perfluoro-1-octanesulfonamide |  |
| ^13^C_3_ CMAmP-6:2 FOSA | ^13^C_3_-6:2 fluorotelomer sulfonamide alkylbetaine |  |
| ^13^C_3_ PFBA | Perfluoro-n-[2,3,4-^13^C_3_]butanoic acid | Injection standard |
| ^13^C_3_ PFPeA | Perfluoro-n-[3,4,5-^13^C_3_]pentanoic acid |  |
| ^13^C_2_ PFHxA | Perfluoro-n-[1,2-^13^C_2_]hexanoic acid |  |
| ^13^C_2_ PFOA | Perfluoro-n-[^13^C_2_]octanoic acid |  |
| ^13^C_5_ PFNA | Perfluoro-n-[^13^C_5_]nonanoic acid |  |
| ^13^C_2_-PFDA | Perfluoro-n-[^13^C_2_]decanoic acid |  |
| ^13^C2 PFUnDA | Perfluoro-n-[1,2,3,4,5,6,7-^13^C_2_]undecanoic acid |  |
| ^18^O_2_-PFHxS | Sodium perfluoro-1-[^18^O_2_]hexanesulfonate |  |
| ^13^C_4_-PFOS | Sodium perfluoro-1-[^13^C_4_]octanesulfonate |  |

Table S3: Target compounds, the MRM transitions and SRM, and corresponding surrogate standard used for quantification.

| Compound | Quantifier m/z | Surrogate standard |
| --- | --- | --- |
| TFA | 113 > 69 | ^13^C_2_ TFA |
| PFPrA | 163 > 119 | ^13^C_3_ PFPrA |
| PFBA | 213 > 169 | ^13^C_4_ PFBA |
| PFPeA | 263 > 219 | ^13^C_5_ PFPeA |
| PFHxA | 313 > 269 | ^13^C_5_ PFHxA |
| PFHpA | 363 > 319 | ^13^C_4_ PFHpA |
| PFOA | 413 > 369 | ^13^C_8_ PFOA |
| PFNA | 463 > 419 | ^13^C_9_ PFNA |
| PFDA | 513 > 469 | ^13^C_6_-PFDA |
| PFUnDA | 563 > 519 | ^13^C_7_ PFUnDA |
| PFDoDA | 613 > 569 | ^13^C_2_ PFDoDA |
| PFTrDA | 663 > 619 | ^13^C_2_ PFDoDA |
| PFTDA | 713 > 669 | ^13^C_2_ PFTDA |
| PFHxDA | 813 > 769 | ^13^C_2_ PFHxDA |
| PFODA | 913 > 869 | ^13^C_2_ PFHxDA |
| TFMS | 149 > 80 | ^13^C_3_-PFBS |
| PFEtS | 199 > 80 | ^13^C_3_-PFBS |
| PFPrS | 249 > 80 | ^13^C_3_-PFBS |
| PFBS | 299 > 99 | ^13^C_3_-PFBS |
| PFPeS | 349 > 99 | ^13^C_3_-PFHxS |
| PFHxS | 399 > 99 | ^13^C_3_-PFHxS |
| PFHpS | 449 > 99 | ^13^C_8_-PFOS |
| PFOS | 499 > 99 | ^13^C_8_-PFOS |
| PFNS | 549 > 99 | ^13^C_8_-PFOS |
| PFDS | 599 > 99 | ^13^C_8_-PFOS |
| PFDoDS | 699 > 99 | ^13^C_8_-PFOS |
| 4:2 FTSA | 327 > 307 | ^13^C_2_-4:2 FTSA |
| 6:2 FTSA | 427 > 407 | ^13^C_2_-6:2 FTSA |
| 8:2 FTSA | 527 > 507 | ^13^C_2_-8:2 FTSA |
| 10:2 FTSA | 627 > 607 | ^13^C_2_-8:2 FTSA |
| 3:3 FTCA | 241 >117 | ^13^C_2_ 6:2 FTCA |
| 5:3 FTCA | 341 > 237 | ^13^C_2_ 6:2 FTUCA |
| 7:3 FTCA | 441 > 337 | ^13^C_2_ 8:2 FTUCA |
| 6:2 FTUCA | 357 > 293 | ^13^C_2_ 6:2 FTUCA |
| 8:2 FTUCA | 457 > 393 | ^13^C_2_ 8:2 FTUCA |
| 10:2 FTUCA | 557 > 493 | ^13^C_2_ 8:2 FTUCA |
| C6/C6 PFPiA | 701 > 401 | ^13^C_2_ PFDoDA |
| C8/C8 PFPiA | 901 > 401 | ^13^C_2_ PFTDA |
| FBSA | 298> 78 | ^13^C_4_ PFBA |
| Me-FBSA | 312 > 112 | ^13^C_4_ PFBA |
| FHxSA | 398 > 78 | ^13^C_3_-PFHxS |
| Me-FHxSA | 412 > 169 | ^2^H_3_-N-MeFOSA |
| HFPO-DA | 285 > 169 | ^13^C_5_ PFHxA |
| PFECHS | 461 > 381 | ^13^C_8_ PFOA |
| 6:2 Cl-PFESA | 531 > 351 | ^13^C_8_-PFOS |
| 8:2 Cl-PFESA | 631>451 | ^13^C_8_-PFOS |
| N-AP-FBSA | 385 > 85 | ^13^C_3_ CMAmP-6:2 FOSA |
| N-AP-PFeSA | 435 > 85 | ^13^C_3_ CMAmP-6:2 FOSA |
| N-AP-FHxSA | 485 > 85 | ^13^C_3_ CMAmP-6:2 FOSA |
| 5:3 FTB | 414> 58 | ^13^C_3_ CMAmP-6:2 FOSA |
| TAmP-FHxSA | 499> 60 | ^13^C_3_ CMAmP-6:2 FOSA |
| N-AP-6:2 FOSA | 513 > 58 | ^13^C_3_ CMAmP-6:2 FOSA |
| OXAmP-6:2 FOSA | 529 > 440 | ^13^C_3_ CMAmP-6:2 FOSA |
| N-AP-FOSA | 585 > 85 | ^13^C_3_ CMAmP-6:2 FOSA |
| CMAmP-6:2 FOSA | 571> 104 | ^13^C_3_ CMAmP-6:2 FOSA |
| FMeSI | 280>147 | ^13^C_3_-PFBS |
| PFEtSI | 380>197 | ^13^C_3_-PFHxS |
| PFBSI | 580>297 | ^13^C_8_-PFOS |
| BF_4_^-^ | 87* | ^13^C_2_ TFA |
| PF_6_^-^ | 145* | ^13^C_2_ TFA |

*BF_4_^-^ and PF_6_^-^ were acquired in selected reaction monitoring mode.

Table S4: Instrumental parameters for UPLC-MS/MS analysis of PFAS in landfill leachate

| UPLC | |
| --- | --- |
| Column temperature | 50 ℃ |
| Flow rate | 0.3 mL/min |
| Injection volume | 10 µL |
| Gradient | |
| Time | %A : %B |
| 0.00 | 99:1 |
| 13.3 | 0:100 |
| 14.0 | 0:100 |
| 14.2 | 99:1 |
| 17.0 | 99:1 |
| MS | |
| Capillary voltage (kV) | 0.8 |
| Cone voltage (V) | 30 |
| Source temperature (℃) | 150 |
| Desolvation temperature (℃) | 400 |
| Cone Gas Flow (L/Hr) | 150 |
| Desolvation Gas Flow (L/Hr) | 800 |

Table S5: Instrumental parameters for SFC-MS/MS analysis of PFAS in landfill leachate

| SFC | |
| --- | --- |
| Column temperature | 50 ℃ |
| Flow rate | 1.2 mL/min |
| Injection volume | 2 µL |
| Gradient | |
| Time | %A : %B |
| 0.00 | 85:15 |
| 5.00 | 65:35 |
| 6.00 | 65:35 |
| 6.01 | 85:15 |
| 8.00 | 85:15 |
| MS | |
| Capillary (kV) | 2.5 |
| Cone (V) | 27 |
| Source Temperature (°C) | 150 |
| Desolvation Temperature (°C) | 350 |
| Cone Gas Flow (L/Hr) | 50 |
| Desolvation Gas Flow (L/Hr) | 650 |

**Results**


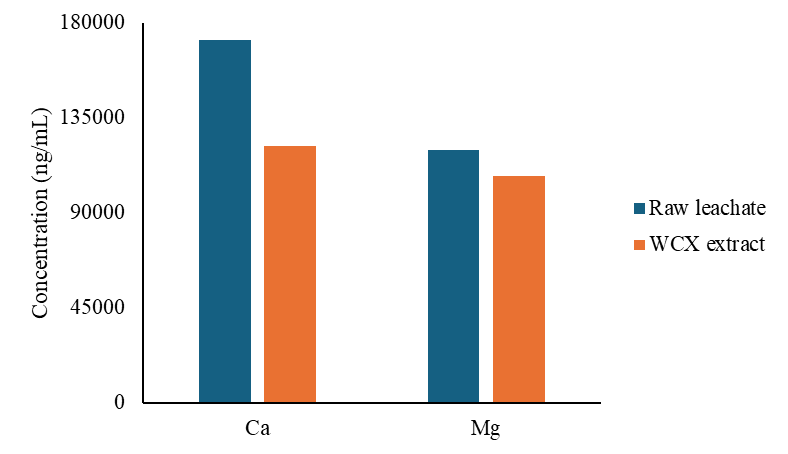


Figure S2: The concentration of magnesium and calcium in raw landfill leachate and leachate after extraction with WCX (n=3).


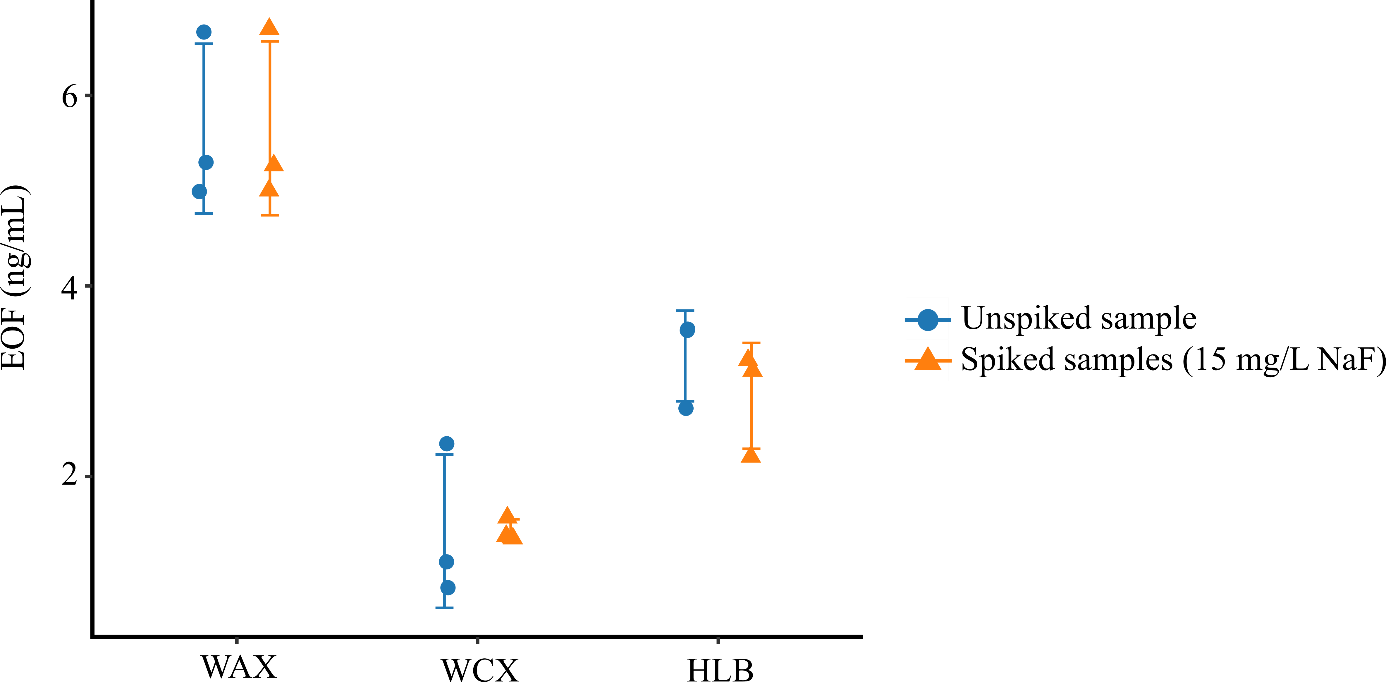


Figure S3: EOF concentrations measured in landfill leachate samples, comparing unspiked samples and samples spiked with NaF prior extraction. Error bars represent the standard deviation of triplicate extractions.


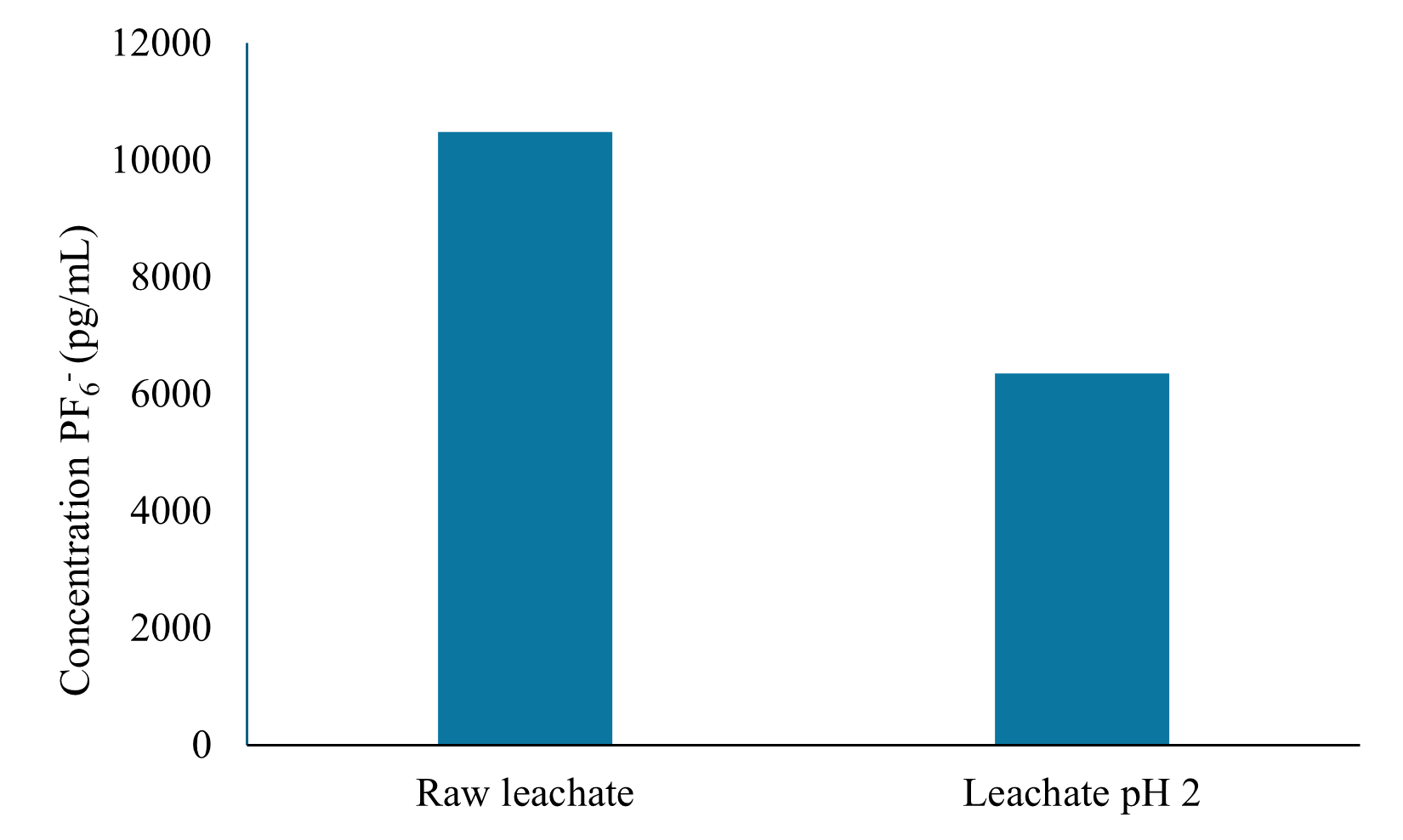


Figure S4: Direct injection of landfill leachate and landfill leachate adjusted to pH 2 with nitric acid

Table S6: Recovery of native standards (n=2) added to landfill leachate

| Class | acronym | SPE-WAX | SPE-WCX | SPE-HLB |
| --- | --- | --- | --- | --- |
| PFCA & PFSA | PFPrA | 61% | 0% | 0% |
|  | TFMS | 59% | 0 | 0% |
|  | PFEtS | 50% | 0% | 0% |
|  | PFPrS | 76% | 0% | 0% |
|  | PFPeS | 94% | 22% | 96% |
|  | PFHpS | 87% | 97% | 97% |
|  | PFNS | 62% | 87% | 69% |
|  | PFDS | 65% | 78% | 65% |
|  | PFDoDS | 35% | 10% | 73% |
| FASA | FBSA | 80% | 31% | 79% |
|  | MeFBSA | 34% | 31% | 2% |
|  | PFHxSA | 17% | 39% | 65% |
|  | MePFHxSA | 1% | 0% | 2% |
| PFPA & PFPiA | PFHxPA | 153% | 152% | 91% |
|  | PFOPA | 42% | 69% | 55% |
|  | 6:6 PFPiA | 35% | 44% | 37% |
|  | 8:8 PFPiA | 50% | 16% | 23% |
| FTCA & FTUCA | 3:3 FTCA | 32% | 0% | 92% |
|  | 5:3 FTCA | 24% | 55% | 70% |
|  | 7:3 FTCA | 8% | 22% | 65% |
| Zwitterionic PFAS | N-AP-FBSA | 73% | 104% | 156% |
|  | N-AP-PFeSA | 101% | 101% | 173% |
|  | N-AP-FHxSA | 101% | 115% | 174% |
|  | 5:3 FTB | 73% | 85% | 96% |
|  | TAmP-FHxSA | 74% | 97% | 100% |
|  | N-AP-6:2 FOSA | 117% | 104% | 271% |
|  | OXAmP-6:2 FOSA | 50% | 84% | 54% |
|  | N-AP-FOSA | 128% | 116% | 50% |
|  | CMAmP-6:2 FOSA | 57% | 90% | 99% |
| Bis-FASI | FMeSI | 17% | 50% | 95% |
|  | PFEtSI | 86% | 94% | 99% |
|  | PFBSI | 57% | 84% | 64% |
| Others | 6:2 Cl-PFESA | 75% | 94% | 84% |
|  | 8:2 Cl-PFESA | 50% | 66% | 59% |
|  | PFECHS | 80% | 92% | 100% |
|  | 6:6 PFPiA | 35% | 44% | 37% |
|  | 8:8 PFPiA | 50% | 16% | 23% |
|  | 10:2 FTSA | 43% | 52% | 64% |
|  | BF_4_^-^ | 37% | 0% | 0% |
|  | PF_6_^-^ | 1% | 5% | 6% |

**References**

[1] Water quality-Determination of perfluoroalkyl and polyfluoroalkyl substances (PFAS) in water-Method using solid phase extraction and liquid chromatography-tandem mass spectrometry (LC-MS/MS). 2019.

[2] Gallen C, Eaglesham G, Drage D, Nguyen TH, Mueller JF. A mass estimate of perfluoroalkyl substance (PFAS) release from Australian wastewater treatment plants. Chemosphere 2018;208:975–83. https://doi.org/10.1016/j.chemosphere.2018.06.024.

[3] Brumovský M, Bečanová J, Karásková P, Nizzetto L. Retention performance of three widely used SPE sorbents for the extraction of perfluoroalkyl substances from seawater. Chemosphere 2018;193:259–69. https://doi.org/10.1016/J.CHEMOSPHERE.2017.10.174.

[4] D’Agostino LA, Mabury SA. Identification of novel fluorinated surfactants in aqueous film forming foams and commercial surfactant concentrates. Environ Sci Technol 2014;48:121–9. https://doi.org/10.1021/es403729e.
